# Killer Timing: The Temporal Uterine Natural Killer Cell Differentiation Pathway and Implications for Female Reproductive Health

**DOI:** 10.3389/fendo.2022.904744

**Published:** 2022-06-27

**Authors:** Rupsha Fraser, Ana Claudia Zenclussen

**Affiliations:** ^1^Centre for Reproductive Health, Queen’s Medical Research Institute, The University of Edinburgh, Edinburgh, United Kingdom; ^2^Department of Environmental Immunology, UFZ-Helmholtz Centre for Environmental Research Leipzig-Halle, Leipzig, Germany

**Keywords:** decidual natural killer (dNK) cells, differentiation, secretory stage endometrium, progesterone, human chorionic gonadotropin, uterine vascular growth, spiral artery remodelling, immune regulation

## Abstract

Natural killer (NK) cells are the predominant maternal uterine immune cell component, and they densely populate uterine mucosa to promote key changes in the post-ovulatory endometrium and in early pregnancy. It is broadly accepted that (a) immature, inactive endometrial NK (eNK) cells in the pre-ovulatory endometrium become activated and transition into decidual NK (dNK) cells in the secretory stage, peri-implantation endometrium, and continue to mature into early pregnancy; and (b) that secretory-stage and early pregnancy dNK cells promote uterine vascular growth and mediate trophoblast invasion, but do not exert their killing function. However, this may be an overly simplistic view. Evidence of specific dNK functional killer roles, as well as opposing effects of dNK cells on the uterine vasculature before and after conception, indicates the presence of a transitory secretory-stage dNK cell (s-dNK) phenotype with a unique angiodevelopmental profile during the peri-implantation period, that is that is functionally distinct from the angiomodulatory dNK cells that promote vessel destabilisation and vascular cell apoptosis to facilitate uterine vascular changes in early pregnancy. It is possible that abnormal activation and differentiation into the proposed transitory s-dNK phenotype may have implications in uterine pathologies ranging from infertility to cancer, as well as downstream effects on dNK cell differentiation in early pregnancy. Further, dysregulated transition into the angiomodulatory dNK phenotype in early pregnancy will likely have potential repercussions for adverse pregnancy outcomes, since impaired dNK function is associated with several obstetric complications. A comprehensive understanding of the uterine NK cell temporal differentiation pathway may therefore have important translational potential due to likely NK phenotypic functional implications in a range of reproductive, obstetric, and gynaecological pathologies.

## Introduction

The uterine mucosa undergoes dynamic cyclical tissue breakdown, regeneration and remodelling, leading to a carefully timed and defined period during which an embryo is able to attach and invade into a receptive uterus (1, 2). In the human endometrium, decidualisation is a dynamic, multistep progression of events, which begins in the secretory stage of the uterine cycle (luteal phase of the menstrual cycle) in response to rising ovarian steroid hormones (progesterone and estradiol) produced by the corpus luteum following ovulation, and is marked by the differentiation of fibroblast-like endometrial stromal cells (ESCs) into large, secretory, ‘decidual’ cells. Implantation of a conceptus takes place in the mid-secretory endometrium during a transient embryo-receptive period known as the ‘window of implantation’ (3). The induction of this receptive endometrial phenotype is reliant on an acute pro-inflammatory decidualisation initiation phase that leads to secretory and phenotypic changes in the uterine epithelium, and is also associated with the accumulation of maternal leukocytes in the endometrium and angiogenic growth of the spiral arteries (the terminating branches of the uterine arteries) in preparation for implantation (3, 4). Decidualisation is maintained after implantation, mainly under the influence of progesterone, and is required for the maintenance of pregnancy (3, 5, 6). In this review, we will focus on events in the human uterus, while referring, where appropriate, to relevant evidence from mouse models.

Innate lymphoid cells (ILCs; lymphocytes that do not express diverse antigen receptors) are the most prevalent leukocytes populating the uterine mucosa during the menstrual cycle and in early pregnancy. ILC subsets are categorised according to their cytokine profiles and transcription factor expression: ILC1s express T-bet and secrete IFN-γ; ILC2s express GATA-3 and secrete IL-5, IL-9 and IL-13; and ILC3s, which include a lymphoid tissue inducer-like subset, are defined by their expression of RORγt and secrete IL-17 and IL-22. Natural killer (NK) cells are known as key members of the ILC family, although it remains to be clarified whether tissue-resident NK cells in the uterine mucosa constitute a unique NK cell population or denote a population that includes ILC1s and ILC3s (7, 8). NK cells represent the predominant maternal immune cell component in the secretory stage endometrium and comprise up to 70% of leukocytes in first trimester decidua: they promote critical changes in the uterine microenvironment both during the ‘window of implantation’ and in early pregnancy (9–11), and uterine NK numbers subsequently diminish from mid-gestation to term (12).

**Figure 1:**
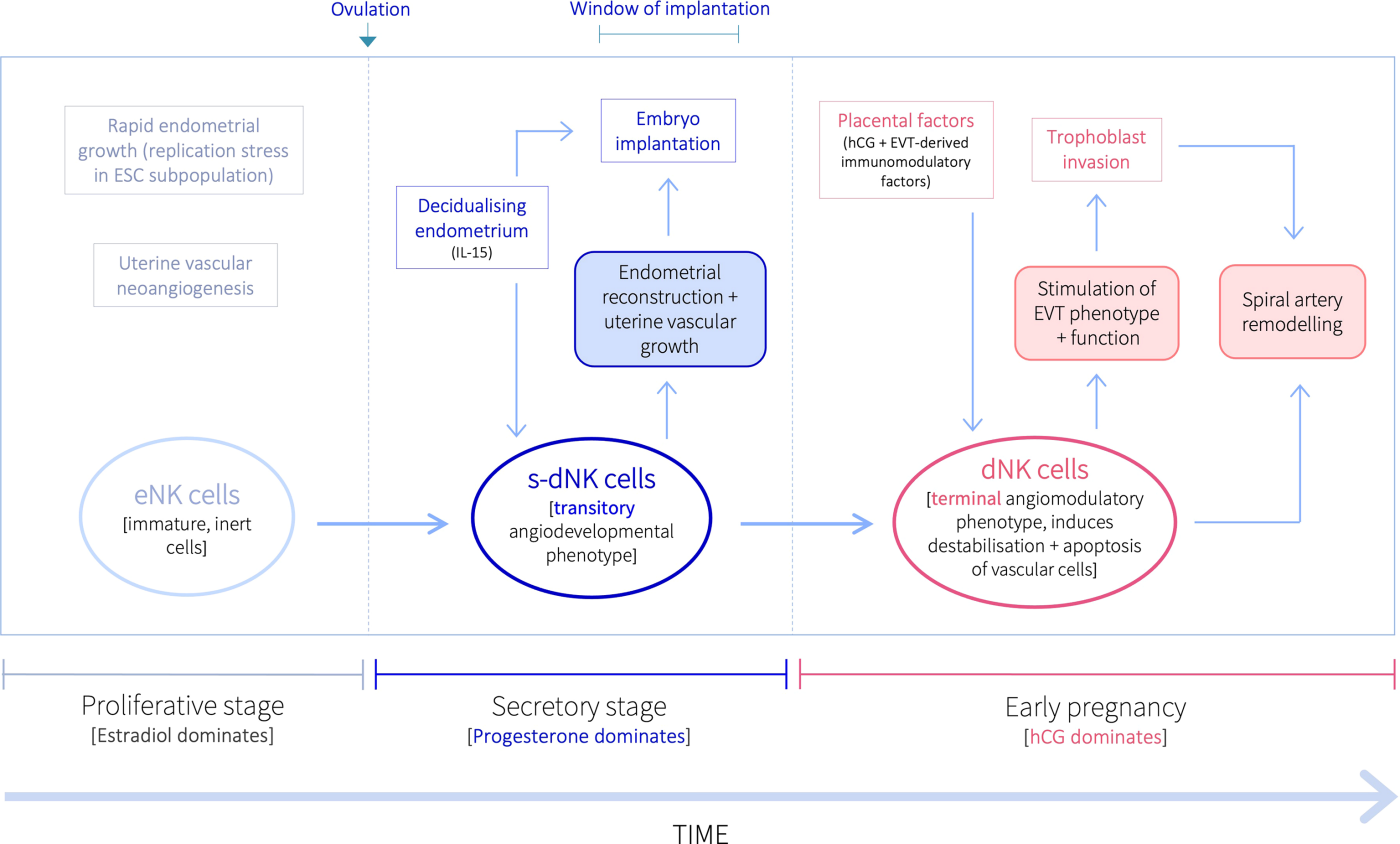
Proposed temporal natural killer cell differentiation pathway within the uterine mucosa. Following ovulation, progesterone upregulates the production of IL-15 by ESCs in the secretory stage endometrium decidualisation progresses. IL-15 stimulates the proliferation, activation, and maturation of inert eNK cells into a transitory angiodevelopmental s-dNK phenotype that induces uterine vascular growth and mediates endometrial reconstruction to facilitate embryo implantation. Following implantation, placental-derived hCG drives further NK proliferation, and EVT-derived immunomodulatory factors promote their differentiation into a terminal angiomodulatory dNK phenotype, with destabilising and pro-apoptotic effects on vascular cells and pro-invasive effects on EVT, to initiate the uterine vascular changes crucial for successful pregnancy outcomes.

Inactive NK cells proliferate and become activated in the decidualising secretory-stage endometrium, and then further expand in pregnancy (13, 14). Recent evidence also suggests that the uterine NK niche may be replenished from the circulation (15). While it was historically believed that the same NK cells that are activated during the secretory stage, expand and continue in pregnancy, evidence from several studies over the last decade indicate progressive NK cell differentiation within the uterine mucosa in response to the local environment during both endometrial regeneration and pregnancy (2, 15–17). However, it is still widely accepted that, despite their name, NK cells in the uterine mucosa do not kill, but are pro-angiogenic (18–22). Activated uterine NK cells do, nevertheless, live up to their name, and induce apoptosis of distinct cellular populations pre- and post-conception, to promote implantation, placentation and uterine vascular remodelling, thus ensuring pregnancy success (9, 10), despite producing several immunosuppressive molecules that may contribute to the establishment of maternal-fetal immune tolerance (23). Uterine NK cells are predominantly CD56^bright^CD16^–^ (as opposed to peripheral blood NK cells, which are largely CD56^dim/–^CD16^bright^), and once activated in the secretory stage, produce cytotoxic proteins and thus have cytolytic capacity (21, 24), but do not cause cytotoxicity of healthy placental cells (22). Further, while secretory-stage dNK cells do indeed stimulate growth and development of the spiral arteries to ensure an adequately vascularised endometrium for implantation (20, 25), dNK cells in early pregnancy bestow destabilising and pro-apoptotic effects on vascular cells to initiate the spiral artery remodelling process requisite for increased vascular provision to the developing fetus (10, 11, 26–30). At this time, uterine NK cells also promote trophoblast invasion, which is when specialised placental-derived cells (trophoblasts) invade the decidua towards the spiral arteries in order to remodel them (10, 31). Further, pre- and post-conception uterine NK cells display different secretory profiles that support distinct uterine vascular processes (25, 26, 29). Thus, uterine NK cells exhibit divergent immune profiles and functions before and after pregnancy, that are both indirectly and directly influenced by the temporal endocrine adaptions within the local uterine tissue environment (9–11, 26, 29, 32–35). Indeed, a recent murine study investigating the early pregnancy uterine transcriptome has demonstrated that endocrine and paracrine regulators of immune responses and different cellular behaviours exhibit differential region-specific expression patterns within the uterus (36), further emphasising the contributory role of the local uterine microenvironment in mediating cellular phenotypic and functional profiles. Here, we describe the disparate functional profiles of NK cells in the uterine mucosa during the ‘window of implantation’ and in early pregnancy. These divergent NK characteristics indicate a pivotal but transitory angiodevelopmental uterine NK cell phenotype that also implements endometrial reconstruction in the peri-implantation endometrium; which is functionally distinct from the equally essential, angiomodulatory uterine NK cell phenotype in early pregnancy that induces vessel structure destablisation, vascular cell apoptosis and trophoblast invasion, to facilitate uterine vascular remodelling and placentation for successful pregnancy outcomes (**Figure 1**).

## Timing of NK Cell Proliferation in the Uterine Mucosa: Secretory Stage vs. Early Pregnancy Expansion

Temporally and physiologically distinct vascular processes during the secretory and early pregnancy are necessary to provide an adequate oxygen and nutrient supply to the placental unit, to meet the demands of a rapidly developing fetus. Successful implantation and establishment of pregnancy rely on uterine vascular development and expansion, whereby decidual capillaries and spiral arteries undergo vascular growth and development, in response to coordinated progesterone and estradiol secretion (20, 37, 38). Subsequently, in early pregnancy, the uterine spiral arteries must be remodelled into large-diameter, high-flow vessels with low resistance, facilitating an approximately 10-fold increase in blood supply into the intervillous space for placental uptake (39). These disparate vascular processes correspond to two separate bursts of uterine NK cell proliferation that are mediated by temporal endocrine and immunological adaptations within the uterine environment: there is a significant increase in number during the peri-implantation period in the secretory stage endometrium, followed by a further increase in early pregnancy (35, 37, 40).

Rapid proliferation of uterine NK cells is concurrent with secretory-stage ESC decidual transformation and growth of the uterine vasculature. Under the action of ovarian steroid hormones, an acute pro-inflammatory ESC response leads to the production of adhesion molecules in the overlying epithelium, which renders the uterus transiently receptive for embryo implantation (18, 41). During this time, the NK cells within the uterine mucosa undergo post-ovulatory phenotypic changes, and display distinctive gene and protein expression profiles, although neither the progesterone receptor (PR) nor estrogen receptors (ERs) are expressed by human uterine mucosal NK cells (32, 42, 43). Instead, the previously inert endometrial NK (eNK) cells are activated by ESC-derived interleukin 15 (IL-15) (35, 42): the mid-secretory endometrium begins to express high levels of IL-15, which is upregulated by progesterone as decidualisation progresses, and promotes their proliferation, activation, and maturation into decidual NK (dNK) cells (44–49). If there is no fertilisation, the corpus luteum will regress, resulting in progesterone (and estradiol) withdrawal, and in turn, IL-15 withdrawal. This leads to death of the activated dNK cells and subsequent decline of dNK-derived soluble factor products that maintain vascular integrity, thereby contributing to spiral artery constriction and endometrial ischemia, and ultimately causing ESC cell death and menses (40, 41, 50, 51). If pregnancy ensues, the uterine microenvironment remains abundantly rich in IL-15 throughout the first trimester of pregnancy, until placentation and uterine vascular remodelling are complete (33, 34). Subsequently, IL-15 levels decline, corresponding to waning dNK numbers from mid-gestation to term (12, 34).

The post-ovulatory dNK cell proliferative surge and activation is explained by increased IL-15 levels in the peri-implantation endometrium. However, the further dramatic dNK cell expansion in early pregnancy remained unexplained until it was demonstrated that extensive post-implantation dNK proliferation can be induced by human chorionic gonadotropin (hCG), one of the earliest proteins secreted by trophoblast cells (35). hCG is a heterodimeric glycoprotein with an identical alpha subunit to luteinizing hormone (LH), and hence binds the LH receptor (LHR); and both mature hCG and LH are modified by N-linked carbohydrate side chains that are important for the stability and assembly of the proteins, although the carbohydrate side chains between the two proteins are not identical (52, 53). Thus, while NK cells in uterine mucosa do not express the LHR, they do express the mannose receptor (MR; also known as CD206), a cell surface lectin that binds glycoproteins with N-linked carbohydrate side chains (54). The uterine NK cell MR can therefore recognise carbohydrate moieties in glycosylated proteins. As such, hCG-carbohydrate-side chain recognition by dNK-expressed MR may induce non-canonical hCG signalling to stimulate dNK proliferation in early pregnancy (35). However, since hCG and LH do not share identical N-linked carbohydrate side chains (53), the pre-ovulatory LH surge (which induces ovulation) is likely unable to promote eNK activation and maturation into dNK cells, as indicated by the lack of uterine NK proliferation reported upon LH stimulation *in vitro* (35). This is also consistent with the significant rise in secretory-stage uterine NK number occurring several days after ovulation, when decidualisation begins. The second surge in dNK cell number, which occurs during early pregnancy and placentation, corresponds to the exponentially rising hCG levels that peak at around 10 weeks of gestation (55); levels then decline until approximately 16^th^ week of gestation and remain relatively constant thereafter until term (55), consistent with declining dNK numbers from mid-gestation to term (12). Notably, failing pregnancies have been associated with lower daily rates of increase in hCG, with low circulating hCG concentrations predictive of pregnancy loss (56). Interestingly, a unique dNK population has been identified in the decidua of multigravid women, termed pregnancy-trained decidual NKs (PTdNKs), whose activation may foster proper placentation, demonstrating the trained memory function of dNK cells in enhancing placentation (57).

## NK Cells in the Secretory Stage Uterine Mucosa

Endometrial neoangiogenesis and rapid endometrial growth take place during the estradiol-mediated proliferative phase of the uterine cycle to regenerate the endometrium after menstruation; and following ovulation, rising levels of progesterone and estradiol initiate the decidualisation process during secretory stage of the uterine cycle (9, 37, 58). Decidualisation is characterised by differentiation of ESCs into specialised decidual cells, NK cell accumulation within the uterine mucosa, uterine vascular growth, and local edema (3, 4). A sufficiently decidualised stroma, with increased vascularisation of the endometrium, is essential for invasion of the blastocyst and embryo implantation (37). Successful implantation in humans also requires a receptive epithelium for initial attachment of the conceptus, which is reliant on a pro-inflammatory endometrial environment (59–64). The presence of NK cells in the endometrium prior to conception is unique to humans, and the functional profiles of secretory-stage dNK cells demonstrate their prominent roles in mediating endometrial reconstruction, promoting growth and development of the uterine vasculature, as well as contributing to an inflammatory endometrial phenotype that is essential for implantation (65).

Decidual transformation is not static, but a dynamic process that begins after ovulation, during the secretory stage of the uterine cycle. It encompasses three critical transitory phases: an acute pro-inflammatory decidualisation initiation phase, which subsequently transitions to an anti-inflammatory secretory phase during which time embryo implantation takes place, followed by a final resolution phase. The transient pro-inflammatory decidualisation initiation response that is crucial for the induction of endometrial receptivity, is accompanied by a surge of free radical production, and the secretion of various cytokines, growth factors and angiogenic factors (4, 61, 66, 67). However, the rapid pre-ovulatory endometrial proliferation (post-menstruation endometrial regeneration) prompts replication stress in a subpopulation of ESCs that are consequently unable to differentiate into specialised decidual cells during the decidualisation initiation phase, but instead undergo acute cellular senescence (a tightly coordinated biological process implicated in embryo development, wound healing and tissue repair) (9). These senescent ESCs, in turn, secrete a host of inflammatory mediators that signal to induce the production of epithelial adhesion molecules involved in endometrial receptivity. Acute decidual senescence, through its stimulation of an acute pro-inflammatory decidual phenotype, thus contributes to the induction of key receptivity gene expression in the overlying endometrial epithelium (9, 61), and may therefore be fundamental to the acquisition of a receptive endometrial phenotype. Concomitant systematic clearance of acutely senescent decidual cells, regulates endometrial reconstruction (rejuvenation and remodelling of ESCs) that is necessary ensure a mature decidual cell environment for embryo implantation. Secretory stage dNK cells are pivotal to this process, as they target and eliminate senescent cells through granule exocytosis. Senescent decidual ESC clearance by midsecretory-stage dNK cells, coincident with the ‘window of implantation’, thereby exerts a role in maintaining tissue homeostasis from cycle to cycle, and may directly assist embryo implantation in the presence of a conceptus (9). In addition, secretory stage dNK cells also release prokineticin 1 during the ‘window of implantation’, which regulates the expression of several implantation-related genes, and has therefore been proposed as a marker of endometrial receptivity (68, 69).

Peri-implantation growth and development of the spiral arteries (branching, elongation and vascular maturation) increases the uterine vascular surface area in preparation for pregnancy, and is prerequisite for implantation and placentation to proceed (37, 70, 71). Secretory-stage dNK cells produce a range of angiogenic mediators including vascular endothelial growth factor (VEGF)-A, VEGF-C, placental growth factor (PlGF), and angiopoietins 1 and 2 (25). The VEGF family constitutes seven distinct proteins, which include VEGF-A, VEGF-C and PlGF. VEGF-A is a key regulator of decidual angiogenesis and the maintenance of vascular integrity, and when genetically ablated in mice, causes embryonic lethality (20, 25, 72). VEGF-C has been characterised as a selective growth factor for lymphatic vessels and was found to affect the migration and proliferation of endothelial cells (25). VEGF signaling is mediated *via* interactions with two structurally related tyrosine kinase receptors, VEGFR-1/fms-like tyrosine kinase I (Flt-1) and VEGFR-2 (Flk-1) (73). Upregulation of VEGF-A and its cognate receptors that act on the uterine microvasculature during the secretory stage, may lead to increased vascularity and blood flow, in turn, enhancing the vascular conditions for implantation (74). PlGF is also heavily expressed in human pregnancies, binds to both Flt-1 and Flk-1, and promotes the further expression of angiogenic factors such as VEGF-A, basic fibroblast growth factor, platelet-derived growth factor beta, matrix metalloproteinases (MMPs) (20, 75). The release of VEGF and PlGF can be further triggered by NK-cell activating receptors NKp30 and NKp44, both of which are expressed by IL-15-activated dNK cells, while inert pre-ovulatory eNK cells lack NKp30 and NKp44 expression (13, 31). These receptors are also upregulated by prolactin (48), levels of which rise in the late secretory stage and are high throughout pregnancy (49). Angiopoietins can also work in collaboration with VEGFs, to control vascular growth and integrity, and can influence the stability of spiral arteries (25). Thus, peri-implantation dNK cells in the secretory stage uterine mucosa have an angiodevelopmental secretome, playing a central role in supporting growth and elongation of the uterine vasculature for subsequent remodelling into enlarged uteroplacental vessels (25, 70).

The profound changes that take place in the secretory stage endometrium are not only important for implantation success, but aberrant decidual transformation can cause endometrial functional inadequacy, which has been implicated in infertility/reproductive disorders, gynaecological disorders, female reproductive tract cancers, and several obstetric complications (4, 28, 62, 76–88). dNK cell functions undoubtedly play fundamental roles during the secretory stage, and any impairment of the immune-endocrine network (including NK cellular functions) at this time could lead to primary reproductive failure, or uterine pathologies such as endometriosis (characterised by the ectopic growth of endometrial tissue) and its associated infertility (69, 89). Further, phenotypic and functional impairment of uterine NK cells (rendering them unable to exert their classical anti-tumour actions) has been demonstrated in endometrial cancer, likely resulting from a dysregulated and imbalanced endocrine-immune network (19, 90).

Abnormal dNK cell activation and differentiation during the secretory stage can also directly interfere with implantation (69). Depending on the extent of dNK functional dysregulation, this may cause implantation failure, or contribute to anomalous elongation of the ‘window of implantation’ to permit abnormal/delayed embryos to implant, and may also lead to downstream repercussions along the continuum of disorders that have their origins in implantation and placental development (including recurrent miscarriage, pre-eclampsia, intrauterine growth restriction and preterm birth). Indeed, recurrent miscarriage has been associated with a significantly altered secretory-stage dNK phenotype compared to fertile women (91). While some studies show no differences in percentage of total secretory-stage dNK cell numbers detected in women with recurrent miscarriage compared to those without, others report increased dNK levels in the endometrium of women with recurrent miscarriage, with further differences in women with primary versus secondary pregnancy loss (91–95). The lack of consensus of these studies indicates a distinct lack of predictive value of secretory-stage dNK numbers for subsequent pregnancy outcome. Secretory-stage dNK phenotypic and functional studies may therefore be more informative.

## NK Cells in the Early Pregnancy Uterine Mucosa

The production of pro-angiogenic factors is fundamental for appropriate vascular development in the peri-implantation uterus and in the very early stages of pregnancy. However, while peri-implantation dNK cells demonstrate vascular constructive functions and have a pro-angiogenic profile with no apoptotic effects on vascular cells (20, 25), anti-angiogenic effects may be required as pregnancy progresses, to permit the physiological change in spiral arteries to take place (26, 29). Spiral arteries consist of several outer layers of vascular smooth muscle cells (VSMC) and an inner layer of endothelial cells (ECs); and spiral artery remodelling involves removal of VSMCs and the replacement of ECs with extravillous trophoblasts (EVTs), which are specialised placental cells arising from the trophectoderm-derived cytotrophoblast. Following implantation, the trophectoderm (the outer layer of the blastocyst) gives rise to the cytotrophoblast, which differentiates along either a villous or an extravillous pathway: villous cytotrophoblasts give rise to the placental villi, across which maternal-fetal gas and nutrient exchange take place; and EVTs migrate and invade through the decidua towards the spiral arteries in order to remodel them. Insufficient spiral artery remodelling and impaired trophoblast invasion have been associated with a range of obstetric complications, recurrent miscarriage, late spontaneous abortion, including pre-eclampsia, intrauterine growth restriction (IUGR), preterm birth, and placental abruption (collectively referred to as the ‘great obstetric syndromes’) (96–101).

Spiral artery remodelling takes place in two stages: an EVT-independent, followed by an EVT-dependent stage. The initial EVT-independent stage of this physiological change in the spiral arteries include VSMC hypertrophy, disorganisation and dedifferentiation, EC activation and vacuolisation, as well as breaks in the VSMC and EC layers: these events take place in the presence of leukocytes, ahead of trophoblast invasion (30, 102–104). Indeed, histological studies of first trimester decidua have detected early apoptotic changes in vascular cells of the spiral artery in the presence of maternal leukocytes but not EVT (103, 104), and VSMC dedifferentiation (30). Further, dNK cells isolated from 9–14-week decidual tissue (from elective termination of normal pregnancies) induced caspase-dependent apoptotic changes in VSMCs and ECs *via* a Fas signalling pathway, while dNK cells isolated from abnormally remodelling pregnancies (with high resistance to blood flow, and therefore at higher risk of developing pre-eclampsia) produced significantly lower levels of pro-apoptotic factors and failed to induce vascular cell apoptosis (10). In addition, dNK cells isolated from normally remodelling first-trimester pregnancies were found to secrete factors that induced endothelial activation and the disruption of endothelial integrity, thus further contributing to the early vascular changes during spiral artery remodelling; whereas dNK cell-secreted factors from abnormally remodelling pregnancies did not promote EC activation and destabilisation (11). Finally, dNK secretion of angiogenic growth factor levels during pregnancy decreases with increasing gestational age (29), and notably, elevated angiogenic factor secretion by dNK cells has been demonstrated in abnormally remodelling pregnancies (26). These lines of evidence demonstrate the essential roles that dNK cells play in initiating vascular remodelling of the maternal uterine spiral arteries (10, 11, 30, 39, 103); and EVT subsequently complete the remodelling process instigated by the dNK cells, to create uteroplacental vessels that provide the fetal blood supply (77, 102, 103, 105). Indeed, anomalous NK cell receptor gene expression has been linked to aberrant remodelling of the uterine vasculature and the primary stage of several human gestational pathologies, and compromised functional capacity of dNK cells in early pregnancy has been associated with recurrent miscarriage and pre-eclampsia, and other disorders that implicate defective spiral artery remodelling (106–110).

In addition to their direct effects on vascular remodelling, dNK cells in early pregnancy also produce a large repertoire of cytokines, growth factors and proteases that can promote EVT motility and invasion (10, 31). For example, dNK cells produce interleukin (IL)-8 and interferon γ-induced protein (IP)-10, while EVTs express their receptors, CXCR-1 and CXCR-3, respectively, which promotes EVT recruitment and migration to the spiral arteries (31). Further, *in vitro* studies demonstrated that dNK cells were able to promote the invasive behaviour of EVTs through a hepatocyte growth factor-mediated mechanism, which was found to be impaired in dNK cells isolated from abnormally remodelling pregnancies at higher risk of developing pre-eclampsia, which also secreted lower levels of several factors with known stimulatory effects on trophoblasts (urokinase plasminogen activator, heparin-binding epidermal growth factor, CXCL16, IL-1β and IL-8) (10). Additionally, as well as directly promoting EVT invasion, dNK cells also influence the differentiation of EVT into an invasive phenotype *via* their secretion of cytokines and chemokines that can alter the EVT adhesion molecule repertoire, whilst also promoting EVT survival (111–114). However, abnormally remodelling pregnancies demonstrate impaired dNK chemoattraction for EVT, as well as a reduced ability to promote EVT differentiation (115). It is noteworthy to add that dNK production of proteases (e.g. matrix metalloproteinases or urokinase plasminogen activator), as well as factors that mediate the upregulation of proteases (IL-1β, IL-6, IL-8, IL-10, leptin and TNF-α), can promote the breakdown of the elastic fibres at the basal sections of decidual spiral arteries and confer changes in the extracellular matrix, to support EVT invasion (116–120). Additionally, dNK-derived VEGF-C may also generate protective effects by conferring EVT resistance to cytotoxicity, as well as stimulating the assembly of EVT into networks of tube-like structures (18, 121). Thus, while dNK promote these EVT phenotypic and functional characteristics that are requisite for successful trophoblast invasion (including EVT survival, differentiation, migration, motility and invasion); EVT, as they migrate and invade through the decidua, influence dNK phenotype and functions, in addition to the placental induction of dNK proliferation through non-canonical hCG signalling. These dNK-trophoblast interactions in early pregnancy therefore represent an interdependent relationship through reciprocal actions, as well as *via* several functional similarities to promote vascular remodelling (27).

dNK cells can interact with invading EVT through their expression of human leukocyte antigen (HLA)-binding cell-surface receptors and that bind to HLA molecules expressed by EVT. For example, EVT-derived membrane-bound HLA-G and its soluble form (sHLA-G; secreted by EVT) have been shown to stimulate NK cell proliferation and cytokine stimulation, to promote uterine vascular modelling (122, 123). Additional actions of the homodimer form of the sHLA-G, *via* binding to KIR2DL4 (one of the killer-cell immunoglobulin (Ig)-like receptors expressed by NK cells), increases dNK secretion of IL-6, IL-8 and TNF-α (124). Upregulation of these cytokines may directly contribute to spiral artery remodelling, as well as having stimulatory effects on trophoblast invasion (11, 31, 111, 112, 120, 125, 126). Further, specific dNK-trophoblast interactions through dNK KIR and fetal EVT HLA-C are of particular importance, as certain KIR–HLA-C combinations have been associated with an increased risk of pre-eclampsia, recurrent miscarriage and IUGR (108–110). The repertoire of KIRs is heterogeneous, with two haplotypes: A and B. The A haplotype has seven KIR loci with only one activating receptor, KIR2SD4; however, despite being an activating receptor, the most common allele of KIR2SD4 has a deletion, resulting in individuals with two A haplotypes having no activating receptors. The B haplotypes are characterised by the incidence of extra loci that are not present on the A haplotype, consisting of activating receptors KIR2SD1, 2, 3, 5 and KIR3SD1, and the inhibitory receptors KIR2DL2 and KIR2DL5. HLA-C expresses both maternal and paternal antigens, resulting in two allotypes, HLA-C1 and HLA-C2 (109, 127). In pregnancies where dNK cells with the AA KIR genotype (lacking most or all of the activating receptors) are paired with EVT expressing the HLA-C2 allotype, there is a high risk of pre-eclampsia development (109). Conversely, dNK cells expressing activating KIRs enhance placentation and confer protection against reproductive and obstetric disorders (108, 128, 129), and altered dNK interactions with fetal HLA-C and HLA-G have been observed in abnormally remodelling pregnancies, which may compromise several components of the spiral artery remodelling process (130).

As stated previously, there is broad consent that NK cells in the uterine mucosa do not kill. However, we have described here the killing functions of dNK cells found both in the secretory stage endometrium and in early pregnancy. Further, accumulating evidence indicates that dNK cells can also kill infected cells in the presence of viruses (131–133). Indeed, *in vitro* studies demonstrated that dNK cells are able to invade trophoblast organ culture, co-localise with placental cells, degranulate and kill virally infected trophoblasts, as well as selectively kill intracellular bacteria in EVT without killing the trophoblast (132–134).

## Peri-Implantation vs. Early Pregnancy dNK Cells: Phenotype and Function

The divergent functional profiles of dNK cells before and after conception demonstrate that the temporal endocrine and immunological fluctuations in the uterine microenvironment strongly influence dNK phenotype. Timely differentiation into appropriate dNK phenotypes within the uterine mucosa during the secretory stage and in early pregnancy are crucial for endometrial reconstruction, implantation, and successful pregnancy outcomes. Indeed, we have described here the numerous reproductive and obstetric disorders, including recurrent implantation failure, recurrent miscarriage, pre-eclampsia, IUGR and preterm birth, that have been associated with the absence of appropriate NK cell activation and function (10, 11, 20, 26, 106–110, 115, 130). Further, impaired dNK functional capacity has also been indicated in several uterine pathologies (19, 69, 89, 90). We therefore propose (i) a transitory angiodevelopmental secretory-stage dNK (s-dNK) cell phenotype during the ‘window of implantation’, whose phenotype and functional roles are fundamental for uterine vascular developmental growth and endometrial reconstruction, under indirect progesterone control. This is followed by an immunomodulatory shift in early pregnancy, stimulated by hCG and other placental-derived immunomodulatory factors, into (ii) a terminal angiomodulatory dNK phenotype with destabilising and pro-apoptotic effects on vascular cells, and pro-invasive effects on EVT, to initiate and support uterine vascular remodelling into enlarged uteroplacental vessels (**Figure 1**).

## Concluding Remarks

It is probable that untimely or abnormal activation and differentiation into the transient angiodevelopmental s-dNK phenotype is implicated in a wide range of reproductive, gynaecological, and obstetric disorders; and disruption of further differentiation in early pregnancy into the appropriate angiomodulatory dNK phenotype with pro-apoptotic effects on vascular cells and pro-invasive effects on EVT, will also have serious implications in several adverse pregnancy outcomes. A comprehensive understanding of the stepwise NK cell temporal differentiation pathway within the uterine mucosa could therefore have substantial translational potential due to the important functional roles of s-dNK and dNK cells in normal vs. complicated pregnancies, as well as in a spectrum of uterine pathologies (10, 11, 49), and warrants further investigation.

## Author Contributions

RF developed the scope and focus of the review. RF and AZ wrote the article. All authors approved the submitted version.

## Conflict of Interest

The authors declare that the research was conducted in the absence of any commercial or financial relationships that could be construed as a potential conflict of interest.

## Publisher’s Note

All claims expressed in this article are solely those of the authors and do not necessarily represent those of their affiliated organizations, or those of the publisher, the editors and the reviewers. Any product that may be evaluated in this article, or claim that may be made by its manufacturer, is not guaranteed or endorsed by the publisher.
